# High-flow nasal cannula versus continuous positive airway pressure in primary respiratory support for preterm infants: A systematic review and meta-analysis

**DOI:** 10.3389/fped.2022.980024

**Published:** 2022-11-21

**Authors:** Keren Luo, Yi Huang, Tao Xiong, Jun Tang

**Affiliations:** Department of Neonatology, West China Women's and Children's Hospital, Sichuan University, Chengdu, China

**Keywords:** high-flow nasal cannula oxygen therapy, continuous positive airway pressure, neonatal respiratory support, meta-analysis, respiratory failure

## Abstract

Respiratory support is crucial for the survival of preterm infants, and High-flow Nasal Cannula Oxygen Therapy (HFNC) and Continuous Positive Airway Pressure (CPAP) are commonly used for neonatal respiratory support. This meta-analysis aimed to compare the effects of HFNC and CPAP in primary respiratory support for preterm infants, to provide evidence-based support for clinical practice. PubMed, Embase, Cochrane Library, ClinicalTrials.gov, CNKI, VIP, WANFANG and SinoMed were searched for eligible studies. The primary outcomes included the incidence of treatment failure and the application of mechanical ventilation. A total of 27 eligible studies with 3,351 participants were included. There was no significant difference in the incidence of respiratory support failure [RR = 1.17, 95%CI (0.88–1.56)] and the application of mechanical ventilation [RR = 1.00, 95%CI (0.84–1.19)] between HFNC group and CPAP group. HFNC resulted in lower rate of air leaks [RR = 0.65, 95%CI (0.46–0.92)], nasal trauma [RR = 0.36, 95%CI (0.29–0.45)] and abdominal distension [RR = 0.39, 95%CI (0.27–0.58)], and later time of mechanical ventilation initiating [SMD = 0.60, 95%CI (0.21–0.99)], less duration of oxygen therapy [SMD = −0.35, 95%CI (−0.68 to −0.02)] and earlier enteral feeding [SMD = −0.54, 95%CI (−0.95 to −0.13)]. Alternative non-invasive respiratory support after initial treatment failure resulted in no difference in the application of mechanical ventilation between the two groups [RR = 0.99, 95%CI (0.52–1.88)]. HFNC might be more effective and safer in primary respiratory support for preterm infants. Using CPAP as a remedy for the treatment failure of HFNC could not avoid intubation. For premature infants with the gestational age <28 weeks, HFNC as primary respiratory support still needs to be further elucidated.

**Systematic Review Registration:** https://www.crd.york.ac.uk/prospero/display_record.php?ID=CRD42022313479, identifier: CRD42022313479.

## Introduction

Respiratory failure is one of the primary causes of mortality in preterm infants in Neonatal Intensive Care Units (NICU), making it mostly important to perform respiratory support timely for the newborns (1). Continuous Positive Airway Pressure (CPAP) is one of the earliest applied and conventional non-invasive respiratory support methods (2), which could reduce the risk of respiratory complications also decreasing mortality and improving neurological prognosis in preterm infants (3, 4). However, CPAP has high skill requirements for nurses as the improper use would lead to adverse outcomes including nasal mucosal injury or necrosis, nasal granuloma, nasal vestibular stenosis, and nasal septum deformation or deletion in infants, and the special caps that needs to be worn for fixation to ensure ventilation effect would add discomfort for infants (2, 5, 6). High-flow Nasal Cannula Oxygen Therapy (HFNC), also known as Heated Humidified High Flow Nasal Cannula (HHHFNC), is a newly emerged non-invasive respiratory support technology and has been increasingly applied in NICU as an alternative to CPAP (1, 7–13). Compared with CPAP, HFNC has several merits in promoting alveolar dilation (14–18), improving gas exchange (14, 19), protecting airway mucosa (20), and reducing respiratory work (19).

Neonatal respiratory distress syndrome (NRDS) is a common respiratory complication in premature infants. Surfactant and non-invasive ventilation are the standard treatment for NRDS. Treatment failure is defined as the need for other forms of respiratory support due to the presence of respiratory acidosis, hypoxemia, severe apnea, etc. (21, 22). In recent years, multiple studies, including randomized controlled trials (RCTs) and systematic reviews that compared the effects of HFNC and CPAP, have yielded conflicting results. Some studies (23–29) suggested that HFNC was as effective as CPAP, while the others (30–34) found that the failure rate for HFNC was higher than that for CPAP.

However, published systematic reviews did not thoroughly summarize the current evidence due to several reasons such as language limitation, and the outcomes included were too limited to fully reflect the therapeutic effect of HFNC/CPAP to be the primary respiratory support approach for preterm infants with NRDS (26, 27, 29, 32–34). On the other hand, there have been some new studies published in recent years. We performed this systematic review and meta-analysis, based on a more comprehensive literature search, to compare the effects of HFNC and CPAP in respiratory support for preterm infants, so as to provide evidence-based medical support for clinical practice.

## Methods

### Study registration

This systematic review and meta-analysis was conducted following the PRISMA statement (35) and has been registered on PROSPERO (Registration No. CRD42022313479) (36).

### Inclusion criteria and exclusion criteria

Randomized controlled trials (RCTs) comparing the effects of CPAP and HFNC in primary respiratory support for preterm infants (Defined as infants with gestational age <37 weeks) were included. Studies with non-RCT-design, incomplete data, or data unavailable were excluded.

### Outcome measures

Primary outcomes included the incidence of treatment failure and application of mechanical ventilation after non-invasive respiratory support. Outcomes of safety included air leaks, nasal trauma and abdominal distension.

Secondary outcomes included age of respiratory failure onset, duration of mechanical ventilation/non-invasive respiratory support/oxygen therapy, time of mechanical ventilation initiating, time of enteral feeding, exclusive breastfeeding, death, length of hospital stay, and report of adverse events such as hypercapnia, apnea, pulmonary hemorrhage, pneumonia, bronchopulmonary dysplasia (BPD), intraventricular hemorrhage (IVH), periventricular leukomalacia, retinopathy of prematurity (ROP), patent ductus arteriosus (PDA), necrotizing enterocolitis (NEC), sepsis, and requirement for other treatment.

### Literature search

PubMed, Embase, Cochrane Library, ClinicalTrials.gov, CNKI, VIP, WANFANG and SinoMed were searched for relevant articles from inception to February 26th, 2022, with no language restriction. Literature search was conducted by two reviewers independently (Luo and Tang). Detailed search strategy is provided in Appendix 1.

### Literature screening and quality assessment

All the retrieved articles were screened through browsing titles and abstracts to exclude ineligible studies. Afterwards, the full-text of remained articles were read to identify studies that should be included.

The Cochrane risk of bias assessment tool was used for assess the quality of included study, which includes the following 7 items: (1) random sequence generation; (2) allocation concealment; (3) blinding of participants and personnel; (4) blinding of outcome assessment; (5) incomplete outcome data; (6) selective reporting; (7) other bias. Each item could be graded as low risk, high risk, and unclear bias.

Literature screening and quality assessment were performed by two reviewers independently (Luo and Tang). Any disagreements were consulted and settled by a third reviewer (Huang and Xiong).

### Data extraction

Data extraction was processed by two reviewers independently (Luo and Tang) using a pre-designed form. Any controversies were resolved through discussion or by a third reviewer.

### Data synthesis and analysis

Revman was used to conduct the data analysis, and STATA was used to assess the publication bias if needed (No. of included studies >10). Standard mean difference (SMD) with the 95% Confidence interval (95%CI) were pooled for continuous data, and risk ratio (RR) with the 95%CI for dichotomous data. A *p* value less than 0.05 with the 95%CI not included the null indicated statistical significance. Heterogeneity was conducted using I² statistics. Random effect model was applied as pooled statistics if *I*² > 50%, otherwise fixed effect model would be applied. Sensitivity analysis was conducted by removing studies with potential heterogeneity.

## Results

### Characteristics of included studies

**Figure 1:**
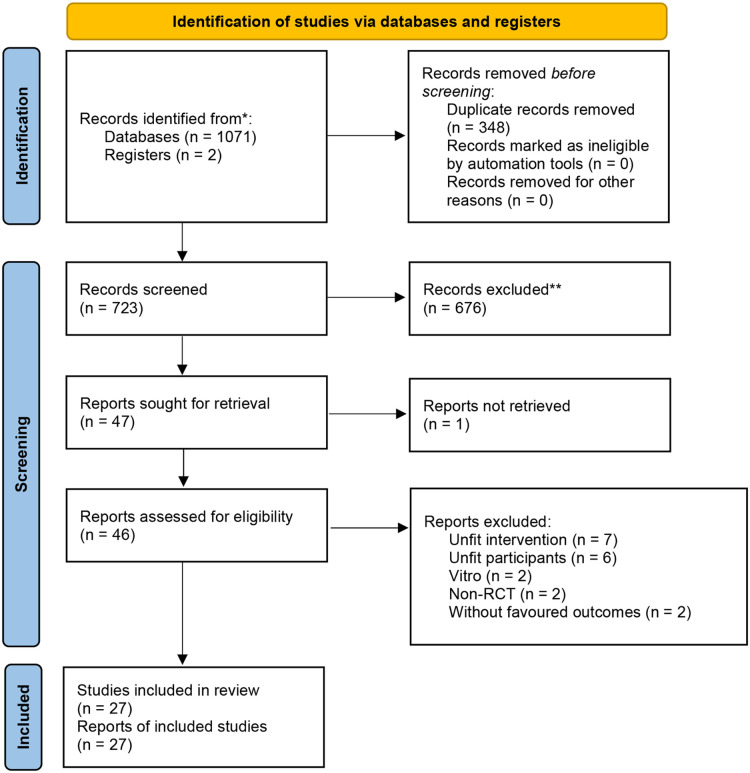
Flow chart.

There was 723 related articles identified. After reading the titles, abstracts and full texts, a total of 27 eligible studies with 3,351 participants (1,664 in HFNC group and 1,687 the CPAP group) were included (23–25, 28, 30, 31, 37–57). The flow diagram for study selection is shown in Figure 1. Among the included studies, 2 included both premature infants and term infants (28, 43), and only data of premature infants was extracted, 2 studies did not specify whether the included preterm infants had NRDS (25, 50), and 3 studies included preterm infants with early respiratory distress, or an intention to respiratory support (44, 48, 57). The participants reported in the other studies were all preterm infants with NRDS. The basic characteristics of included studies is shown in Table 1.

**Table 1:** Characteristics of included studies.

Author	Year	Region	Study design	Gestational age	Comparison
Armanian	2019	Iran	RCT	<37 weeks	HHHFNC (2.5–3 L/min, *n* = 35); nCPAP (5–6 cm H_2_O, *n* = 37)
Chen	2015	China	RCT	<37 weeks	HHHFNC (2–8 L/min, *n* = 34); nCPAP (5–7 cm H_2_O, *n* = 32)
Ciuffini	2014	Italy	RCT	29–36 weeks	HHHFNC (4–6 L/min, *n* = 85); nCPAP (4–6 cm H_2_O, *n* = 92)
Demirel	2019	Turkey	RCT	<32 weeks	HHHFNC (6–8 L/min, *n* = 53); nCPAP (6–7 cm H_2_O, *n* = 54)
Farhat	2018	Iran	RCT	28–34 weeks	HHHFNC (2–5 L/min, *n* = 54); nCPAP (6–8 cm H_2_O, *n* = 53)
Feng	2016	China	RCT	<37 weeks	HHHFNC (*n* = 62); nCPAP (*n* = 68) “detailed parameters” was not provided.
Kadivar	2016	Iran	RCT	28–34 weeks	HHHFNC (2–4 L/min, *n* = 27); nCPAP (5–8 cm H_2_O, *n* = 27)
Lavizzari	2016	Italy	RCT	29–36weeks	HHHHFNC (4–6 L/min, *n* = 158); nCPAP (4–6 cm H_2_O, *n* = 158)
Li	2014	China	RCT	<37 weeks	HHHFNC (6–8 L/min, *n* = 21); nCPAP (4–6 cm H_2_O, *n* = 20)
Manley	2019	Australia	RCT	31–34 weeks	HHHFNC (6–8 L/min, *n* = 72); nCPAP (6–8 cm H_2_O, *n* = 68)
Mostafa-	2015	Iran	RCT	30–34 weeks	HHHFNC (6 L/min, *n* = 42); nCPAP (5–6 cm H_2_O, *n* = 43)
Murki	2018	India	RCT	28–37 weeks	HHHFNC (5–7 L/min, *n* = 133); nCPAP (5–7 cm H_2_O, *n* = 139)
Öktem	2021	Turkey	RCT	<32 weeks	HHHFNC (initial 5 L/min, *n* = 20); nCPAP (5–6 cm H_2_O, *n* = 20)
Roberts	2016	Australia and Norway	RCT	28–37 weeks	HFNC (6–8 L/min, *n* = 278); CPAP (6–8 cm H_2_O, *n* = 286)
Sharma	2019	India	RCT	26–34 weeks	HHHFNC (*n* = 50); nCPAP (*n* = 50) “detailed parameters” was not provided.
Shin	2017	Korea	RCT	30–35weeks	HHHFNC (3–7 L/min, *n* = 42); nCPAP (4–7 cm H_2_O, *n* = 43)
Shirvani	2020	Iran	RCT	<34 weeks	HHHFNC (3–7 L/min, *n* = 30); nCPAP (4–6 cm H_2_O, *n* = 30)
Shokouhi	2019	Iran	RCT	28–36 weeks	HHHFNC (2–8 L/min, *n* = 30); nCPAP (4–8 cm H_2_O, *n* = 30)
Wang	2013	China	RCT	<32 weeks	HHHFNC (2–8 L/min, *n* = 30); nCPAP (4–8 cm H_2_O, *n* = 30)
Wang	2021	China	RCT	28–32 weeks	HHHFNC (3–8 L/min, *n* = 62); nCPAP (3–8 cm H_2_O, *n* = 63)
Yan	2020	China	RCT	<37 weeks	HHHFNC (2–8 L/min, *n* = 47); nCPAP (4–8 cm H_2_O, *n* = 47)
Yao	2019	China	RCT	<35 weeks	HHHFNC (6–8 L/min, *n* = 47); nCPAP (5–7 cm H_2_O, *n* = 47)
Yoder	2013	US	RCT	28–32 weeks	HHHFNC (3–8 L/min, *n* = 75); nCPAP (5–8 cm H_2_O, *n* = 75)
Yu	2018	China	RCT	<36 weeks	HHHFNC (1–7 L/min, *n* = 55); nCPAP (*n* = 55)
Zhai	2019	China	RCT	28–37 weeks	HHHFNC (4–6 L/min, *n* = 38); nCPAP (4–6 cm H_2_O, *n* = 35)
Zhang	2017	China	RCT	<37 weeks	HHHFNC (2–8 L/min, *n* = 44); nCPAP (5–7 cm H_2_O, *n* = 45)
Zhang	2019	China	RCT	<37 weeks	HHHFNC (2–8 L/min, *n* = 40); nCPAP (5–7 cm H_2_O, *n* = 40)

HFNC, high-flow nasal cannula; HHHFNC, heated humidified high flow nasal cannula; CPAP, continuous positive airway pressure; nCPAP, nasal continuous positive airway pressure.

### Risk of bias assessment for included studies

**Figure 2:**
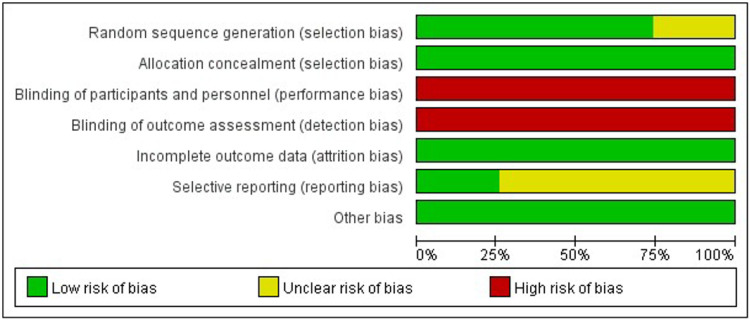
Risk of bias graph: review authors’ judgements about each risk of bias item presented as percentages across all included studies.

**Figure 3:**
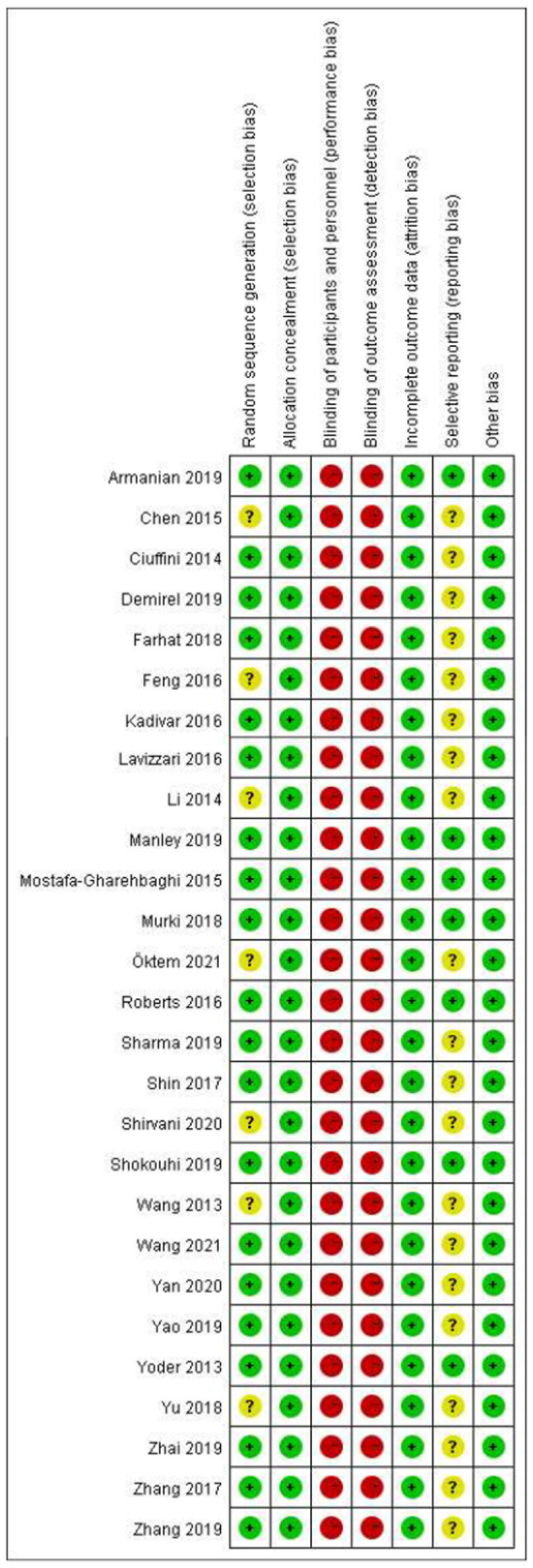
Risk of bias summary: review authors’ judgements about each risk of bias item for each included study.

All studies were RCT-design, while the detailed randomization process was not described in 7 studies (24, 40, 42, 46, 48, 50, 54). All the studies reported allocation concealment, but no blinding of participants and personnel or blinding of outcome assessment. Another factor that might compromise the quality of included studies was that few of them (23–25, 30, 38–42, 46–48, 50–57) provided materials to ensure no reporting bias existed (Figures 2, 3, Appendix 2).

### Primary outcomes

#### Incidence of treatment failure

**Figure 4:**
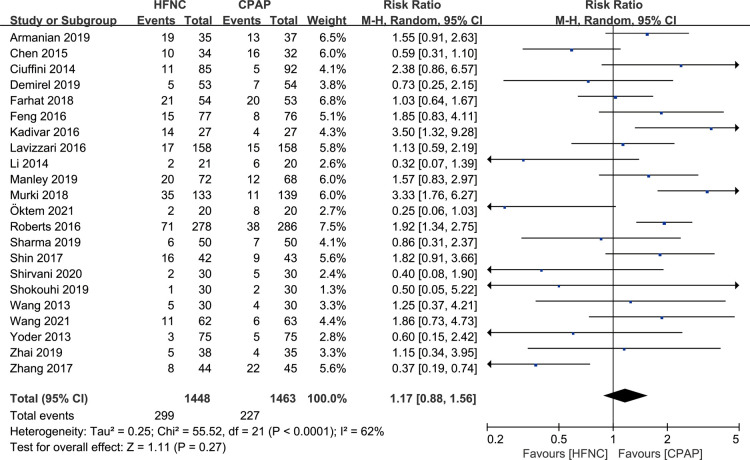
Forest plot of comparison: HFNC vs. CPAP-number-respiratory support failure.

There were 22 studies that reported the incidence of respiratory support failure. Meta-analysis based on random effect model (*I*^2^ = 62%) showed that there was no significant difference in the incidence of respiratory support failure between HFNC group and CPAP group [RR = 1.17, 95%CI (0.88–1.56)], as shown in Figure 4.

#### Application of mechanical ventilation

**Figure 5:**
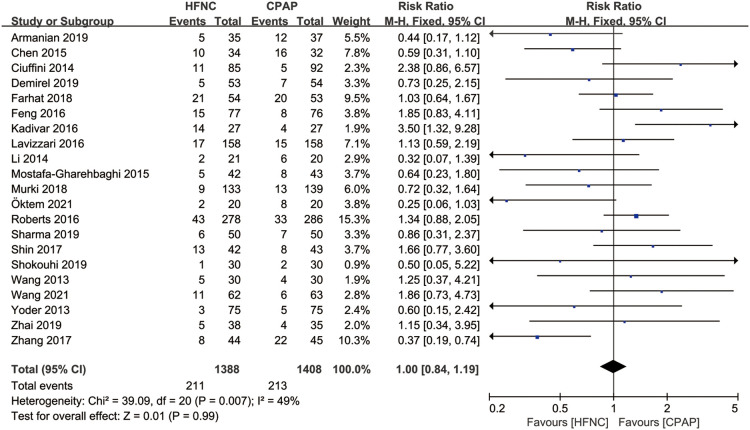
Forest plot of comparison: HFNC vs. CPAP-number-mechanical ventilation.

There were 21 studies that reported the application of mechanical ventilation after non-invasive respiratory support. Meta-analysis based on fixed effect model (*I*^2^ = 49%) showed that there was no significant difference in the application of respiratory support failure between HFNC group and CPAP group [RR = 1.00, 95%CI (0.84–1.19)], as shown in Figure 5.

### Outcomes of safety

**Figure 6:**
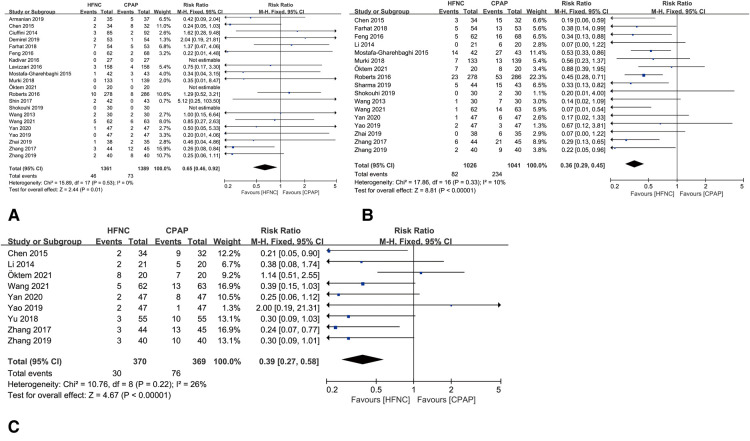
(**A**) forest plot of comparison: HFNC vs. CPAP-number-Air leaks. (**B**) Forest plot of comparison: HFNC vs. CPAP-number-Nasal trauma. (**C**) Forest plot of comparison: HFNC vs. CPAP-number-Abdominal distention.

Compared with CPAP group, HFNC resulted lower rate of air leaks [RR = 0.65, 95%CI (0.46–0.92), *I*^2^ = 0%], nasal trauma [RR = 0.36, 95%CI (0.29–0.45), *I*^2^ = 10%] and abdominal distension [RR = 0.39, 95%CI (0.27–0.58), *I*^2^ = 26%], as shown in Figures 6A–C.

### Secondary outcomes

**Figure 7:**
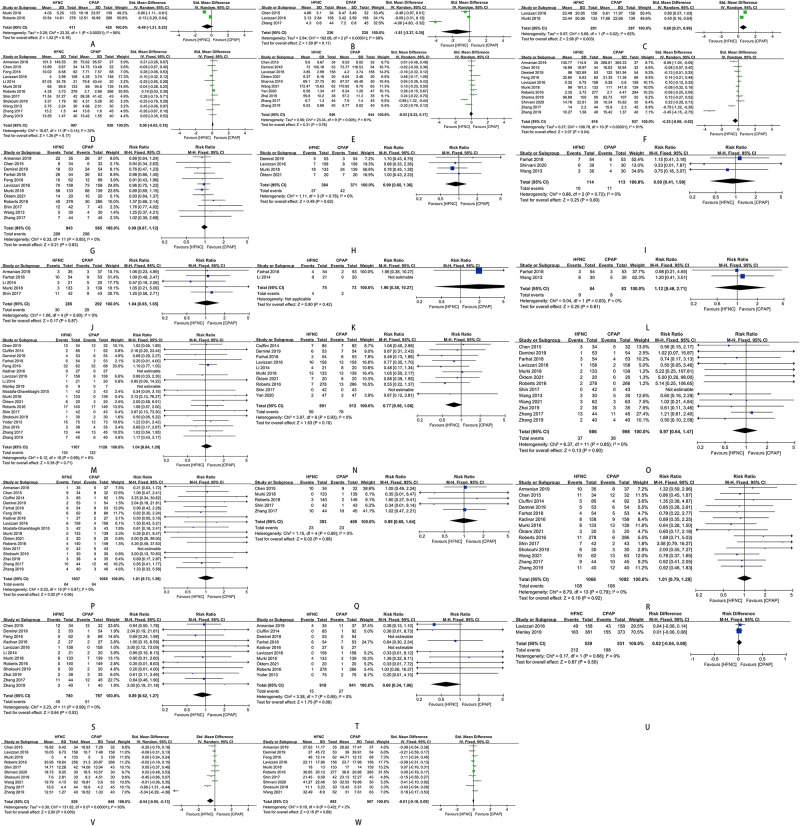
(**A**) forest plot of comparison: HFNC vs. CPAP-age-respiratory failure onset. (**B**) Forest plot of comparison: HFNC vs. CPAP-duration-Mechanical ventilation. (**C**) Forest plot of comparison: HFNC vs. CPAP-age-Mechanical ventilation initiating. (**D**) Forest plot of comparison: HFNC vs. CPAP-duration-Respiratory support. (**E**) Forest plot of comparison: HFNC vs. CPAP-duration-Non-invasive respiratory support. (**F**) Forest plot of comparison: HFNC vs. CPAP-duration-Oxygen therapy. (**G**) Forest plot of comparison: HFNC vs. CPAP-number-Surfactant. (**H**) Forest plot of comparison: HFNC vs. CPAP-number-Surfactant with multiple doses. (**I**) Forest plot of comparison: HFNC vs. CPAP-number-Hypercapnia. (**J**) Forest plot of comparison: HFNC vs. CPAP-number-Apnea. (**K**) Forest plot of comparison: HFNC vs. CPAP-number-Pulmonary hemorrhage. (**L**) Forest plot of comparison: HFNC vs. CPAP-number-Pneumonia. (**M**) Forest plot of comparison: HFNC vs. CPAP-number-Bronchopulmonary dysplasia. (**N**) Forest plot of comparison: HFNC vs. CPAP-number-Sepsis. (**O**) Forest plot of comparison: HFNC vs. CPAP-number-Necrotizing enterocolitis. (**P**) Forest plot of comparison: HFNC vs. CPAP-number-Intraventricular hemorrhage. (**Q**) Forest plot of comparison: HFNC vs. CPAP-number-Periventricular leukomalacia. (**R**) Forest plot of comparison: HFNC vs. CPAP-number-Patent ductus arteriosus. (**S**) Forest plot of comparison: HFNC vs. CPAP-number-Retinopathy of prematurity. (**T**) Forest plot of comparison: HFNC vs. CPAP-number-Death. (**U**) Forest plot of comparison: HFNC vs. CPAP-number-Exclusive breastfeeding. (**V**) Forest plot of comparison: HFNC vs. CPAP-age-Enteral feeding. (**W**) Forest plot of comparison: HFNC vs. CPAP-length-Hospital stay.

The time of mechanical ventilation initiating in preterm infants was later in HFNC group than in CPAP group [SMD = 0.60, 95%CI (0.21–0.99), *I*^2^ = 82%] (Figure 7C). The duration of oxygen therapy in preterm infants in HFNC group was less than those in CPAP group [SMD = −0.35, 95%CI (−0.68 to −0.02), *I*^2^ = 91%] (Figure 7F). The time of enteral feeding in preterm infants in HFNC group was earlier than those in CPAP group [SMD = −0.54, 95%CI (−0.95 to −0.13), *I*^2^ = 93%] (Figure 7V). Other outcomes did not show any statistically significant differences between the two groups: age of respiratory failure onset (Figure 7A), duration of mechanical ventilation (Figure 7B), duration of respiratory support (Figure 7D)/non-invasive respiratory support (Figure 7E), time of exclusive breastfeeding (Figure 7U), death (Figure 7T), length of hospital stay (Figure 7W), and adverse events of hypercapnia (Figure 7I), apnea (Figure 7J), pulmonary hemorrhage (Figure 7K), pneumonia (Figure 7L), BPD (Figure 7M), sepsis (Figure 7N), NEC (Figure 7O), IVH (Figure 7P), periventricular leukomalacia (Figure 7Q), PDA (Figure 7R), ROP (Figure 7S), and requirement for other treatment (Figures 7G,H).

### Sensitivity analysis

**Figure 8:**
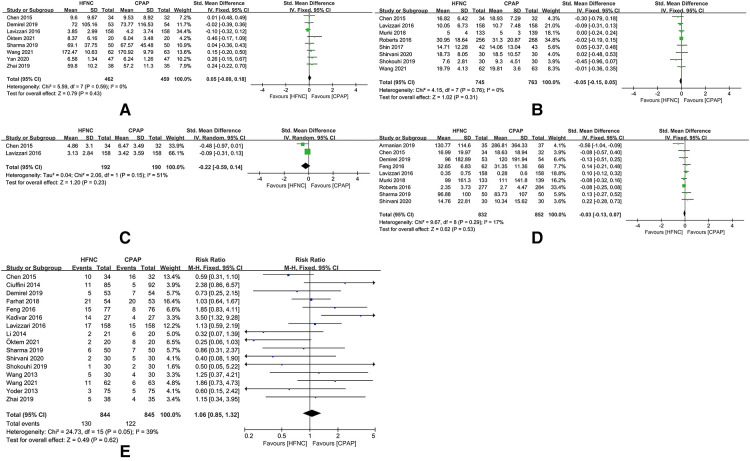
(**A**) Forest plot of comparison: HFNC vs. CPAP-duration-Non-invasive respiratory support (after removing “Zhang 2017” and “Zhang 2019”). (**B**) Forest plot of comparison: HFNC vs. CPAP-age-Enteral feeding (after removing “Zhang 2017” and “Zhang 2019”). (**C**) Forest plot of comparison: HFNC vs. CPAP-duration-Mechanical ventilation (after removing “Zhang 2017”). (**D**) Forest plot of comparison: HFNC vs. CPAP-duration-Oxygen therapy (after removing “Zhang 2017” and “Zhang 2019”). (**E**) Forest plot of comparison: HFNC vs. CPAP-number-Respiratory support failure (after removing “Armanian 2019”, “Manley 2019”, “Murki 2018”, “Roberts 2016”, “Shin 2017”, and “Zhang 2017”).

After removing the studies that increase heterogeneity of this meta-analysis, there were significant differences in the duration of non-invasive respiratory support [SMD = 0.05, 95%CI (−0.08 to 0.18), *I*^2^ = 0%], time of enteral feeding [SMD = −0.05, 95%CI (−0.15 to 0.05), *I*^2^ = 0%], duration of mechanical ventilation [SMD = −0.22, 95%CI (−0.59 to 0.14), *I*^2^ = 51%], duration of oxygen therapy [SMD = −0.03, 95%CI (−0.13 to 0.17), *I*^2^ = 17%), and incidence of respiratory support failure [RR = 1.06, 95%CI (0.85–1.32), *I*^2^ = 39%) (Figures 8A–E).

**Figure 9:**
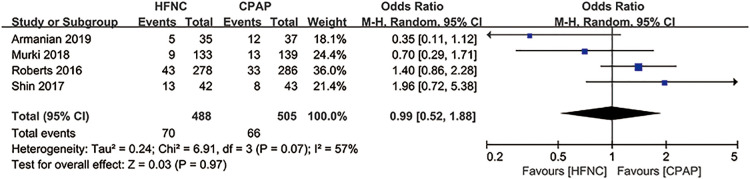
Forest plot of comparison: HFNC vs. CPAP-number-Mechanical ventilation in the studies using alternative non-invasive respiratory support as a remedy for treatment failure.

In five studies (30, 31, 37, 43, 45), alternative non-invasive respiratory support was used as a remedy for treatment failure (Table 2). Since the data of mechanical ventilation for preterm infants were not provided in “Manley 2019” (43), we evaluated the application of mechanical ventilation in the other four studies, and found that alternative non-invasive respiratory support after initial treatment failure resulted in no difference in the application of mechanical ventilation between HFNC group and CPAP group [RR = 0.99, 95%CI (0.52–1.88), *I*^2^ = 57%] (Figure 9).

**Table 2:** Alternative non-invasive respiratory support was used as a remedy for treatment failure.

Author	Year	Respiratory support when HFNC/CPAP fails
Armanian	2019	From HFNC/CPAP to NIMV, NCPAP, or MV.
Manley	2019	From HFNC to CPAP/MV and from CPAP to MV.
Murki	2018	From HFNC to CPAP/MV and from CPAP to MV.
Roberts	2016	From HFNC to CPAP/MV and from CPAP to MV.
Shin	2017	From HFNC to CPAP/MV and from CPAP to Bilevel CPAP/MV.

HFNC, high-flow nasal cannula; CPAP, continuous positive airway pressure; NCPAP, nasal continuous positive airway pressure; NIMV, nasal intermittent mandatory ventilation; MV, mechanical ventilation.

## Discussion

Among the two systematic reviews (32, 34) that were published previously to compare HFNC and CPAP for preterm respiratory distress, one (32) suggested that the both had similar treatment failure rates, while the other (34) proposed that the former resulted in a higher treatment failure rate. In comparison, our study has the following advantages. First, the cut-off years for the included studies were updated, and the search for articles published in Chinese was more comprehensive. Second, this study analyzed multiple respiratory indicators which the previous studies did not included, like duration of mechanical ventilation/non-invasive respiratory support/oxygen therapy, age to use mechanical ventilation, etc. Third, we evaluated the potential of CPAP to be a remedy to avoid intubation when HFNC failed.

In the sensitivity analysis, removing “Zhang 2017” (56) and “Zhang 2019” (57) eliminated the heterogeneity among studies, and the results of the duration of oxygen therapy, duration of mechanical ventilation, duration of non-invasive respiratory support, and time of enteral feeding were reversed, which indicated that there might be bias in these two studies, such as reporting bias due to the lack of protocols, and other undetected bias. In “Armanian 2019” (37), “Manley 2019” (43), “Murki 2018” (45), “Roberts 2016” (31) and “Shin 2017” (30), preterm infants with treatment failure were not all intubated and mechanically ventilated. Some were replaced with non-invasive respiratory support. The analysis for the incidence of mechanical ventilation showed no difference, indicating that there might be no difference in the use of non-invasive respiratory support approaches between the two groups, and that it might be because the included studies did not apply an unified applicable standard for the use of non-invasive respiratory support. For the incidence of respiratory support failure, I² decreased from 62% to 39% after removing “Armanian 2019” (37), “Manley 2019” (43), “Murki 2018” (45), “Roberts 2016” (31), “Shin 2017” (30), and “Zhang 2017” (56).

In our meta-analysis, incidence of respiratory support failure and mechanical ventilation were adopted to evaluate the efficacy of HFNC and CPAP as primary respiratory support for preterm infants and the results showed no statistical difference. This is consistent with the results of previous studies. Several RCTs and meta-analysis found that HFNC had similar efficacy and safety with CPAP in the initial treatment of neonates with NRDS. There was no significant difference between HFNC and CPAP in the intubation rate and other serious complications (23–29). However, a growing number of studies in recent years have produced conflicting results. An RCT comparing HFNC and CPAP as the initial treatment for preterm infants with NRDS found that although HFNC had no significant difference in complications compared with CPAP, it had a higher failure rate (30). Several RCTs and systematic reviews found that HFNC had a significantly higher therapeutic failure rate than CPAP when used as early respiratory support for neonates with NRDS (31, 33, 34), which indicated that HFNC might be not suitable for the primary respiratory support for preterm infants, and such conclusion might be related to the fact that there was a gap between the nasal prong and the nasal cavity during HFNC treatment, thus the airway pressure could not be well controlled. Both the HFNC and CPAP could provide positive pressure to help delating the airway at an oxygen flow rate over 2l/min, while the pressure provided by HFNC could be affected by the size of nasal prong, gas flow, trachea diameter, air leakage, and the body weight of the newborns, making it difficult to precisely evaluate the pressure generated by HFNC (58).

This study showed that HFNC had a lower risk for air leaks, nasal trauma and abdominal distension than CPAP, suggesting a better safety in HFNC. The lower risk for nasal trauma in the use of HFNC might be related to that the nasal prong does not close the nasal cavity completely, which prevents compression of the skin around the nose, and heated and humidified air flow can reduce the incidence of nasal mucosal injury and bleeding so that to increase the comfort of infants (20). Studies (59, 60) showed that HFNC produced lower positive airway pressure than CPAP. An animal experiment found that HFNC produced airway pressure of approximately 3–3.5 cm H_2_O at a flow rate of 6 L/min (60). The above reasons could explain the lower incidence of air leaks and abdominal distension in HFNC compared with CPAP.

Analyses for secondary outcomes showed that the time of mechanical ventilation initiating for preterm infants was later in HFNC group than in CPAP group, which might be associated with the operating mechanism of HFNC. Compared with CPAP, the gas flow rate of HFNC can produce a positive end-expiratory pressure to promote lung expansion and improve alveolar distension (15–18). Also, high-flow gas can flush the anatomic dead cavity in nasopharynx, contributing to the removal of carbon dioxide and the improvement of gas exchange (14, 19). The air flow provided by HFNC exceeds the patient's maximum inspiratory flow, which can minimize the inspiratory resistance of the upper respiratory tract and reduce the work of breath (19). The lower incidence of abdominal distension in HFNC group might be related to earlier attainment of enteral feeding.

Two of the included studies analyzed ultra-premature infants under 28 weeks of gestation. In “Demirel 2019” (25), no significant difference of efficacy and safety was found between HFNC group and CPAP group for ultra-premature infants. In “Öktem 2021” (46), the intubation rate was higher in CPAP group than in HFNC group for the same population (60% vs. 15%, *p* = 0.02). “Sharma 2019” (47) included infants with the gestational age of 26–34 weeks, but did not provide the detailed characteristics of participants. The other included studies did not specifically report whether there were newborns with the gestational age of less than 28 weeks.

However, there are limitations in our meta-analysis. First, there were differences in the baseline characteristics of the included neonates, such as gestational age, birth weight, concomitant NRDS, the severity of NRDS, flow rate of HFNC, pressure of CPAP, which might lead to heterogeneity among studies. Second, due to the lack of specific data on individuals, subgroup analysis based on gestational age or birth weight could not be performed. Third, the included studies did not report all the outcomes in our meta-analysis, which might affect the robustness of the results. Forth, we have expressed there is 25% selection bias and 75% reporting bias. Among them, blinding of participants and personnel or blinding of outcome assessment are unavoidable because the subjects of the study are infants. Furthermore, among the RCTs included, studies from China accounted for the largest proportion, so the applicability of the conclusions in other regions needs to be further verified.

## Conclusion

Compared with CPAP, the use of HFNC for preterm infants might be more effective in reducing the use of mechanical ventilation and oxygen therapy, and has lower risks for air leaks, nasal trauma and abdominal distension. Using CPAP as a remedy for the treatment failure of HFNC could not avoid intubation. For premature infants with the gestational age less than 28 weeks, the use of HFNC as the primary respiratory support still needs to be further elucidated.

## Data Availability

The original contributions presented in the study are included in the article/**Supplementary Material**, further inquiries can be directed to the corresponding author/s.

## References

[1] Motojima Y Ito M Oka S Uchiyama A Tamura M Namba F. Use of high-flow nasal cannula in neonates: nationwide survey in Japan. Pediatr Int. (2016) 58(4):308–10. 10.1111/ped.12903 27095676

[2] Manley BJ. Nasal high-flow therapy for preterm infants: review of neonatal trial data. Clin Perinatol. (2016) 43(4):673–91. 10.1016/j.clp.2016.07.005 27837752

[3] Alallah J. Early CPAP versus surfactant in extremely preterm infants. J Clin Neonatol. (2012) 1(1):12–3. 10.4103/2249-4847.92233 24027675 PMC3761985

[4] Lissauer T Duke T Mellor K Molyneux L. Nasal CPAP for neonatal respiratory support in low and middle-income countries. Arch Dis Child Fetal Neonatal Ed. (2017) 102(3):F194–F6. 10.1136/archdischild-2016-311653 28219880

[5] Jatana KR Oplatek A Stein M Phillips G Kang DR Elmaraghy CA. Effects of nasal continuous positive airway pressure and cannula use in the neonatal intensive care unit setting. Arch Otolaryngol Head Neck Surg. (2010) 136(3):287–91. 10.1001/archoto.2010.15 20231649 PMC3740519

[6] Li SN Li L Li CL Zhou SP Lu WC. The safety and effectiveness of heated humidified high-flow nasal cannula as an initial ventilation method in the treatment of neonatal respiratory distress syndrome: a protocol for systematic review and meta-analysis. Medicine. (2020) 99(46):e23243. 10.1097/MD.0000000000023243 33181713 PMC7668501

[7] Rodriguez Losada O Montaner Ramón A Gregoraci Fernández A Flores España V Gros Turpin A Comuñas Gómez JJ Use of high flow nasal cannula in spanish neonatal units. Anales de Pediatria. (2021) 96:319–25. 10.1016/j.anpedi.2021.02.012 35523688

[8] Hosheh O Edwards CT Ramnarayan P. A nationwide survey on the use of heated humidified high flow oxygen therapy on the paediatric wards in the UK: current practice and research priorities. BMC Pediatr. (2020) 20(1):109. 10.1186/s12887-020-1998-1 32138701 PMC7059285

[9] Petrillo F Gizzi C Maffei G Matassa PG Ventura ML Ricci C Neonatal respiratory support strategies for the management of extremely low gestational age infants: an Italian survey. Ital J Pediatr. (2019) 45(1):44. 10.1186/s13052-019-0639-5 30971298 PMC6458627

[10] Eklund WM Scott PA. High-Flow nasal cannula practice patterns reported by neonatologists and neonatal nurse practitioners in the United States. Adv Neonatal Care. (2018) 18(5):400–12. 10.1097/ANC.0000000000000536 30063474

[11] Schmid F Olbertz DM Ballmann M. The use of high-flow nasal cannula (HFNC) as respiratory support in neonatal and pediatric intensive care units in Germany - A nationwide survey. Respir Med. (2017) 131:210–4. 10.1016/j.rmed.2017.08.027 28947032

[12] Mukerji A Shah PS Shivananda S Yee W Read B Minski J Survey of noninvasive respiratory support practices in Canadian neonatal intensive care units. Acta Paediatrica. (2017) 106(3):387–93. 10.1111/apa.13644 27783410

[13] Roberts CT Owen LS Manley BJ Davis PG. High-flow support in very preterm infants in Australia and New Zealand. Arch Dis Child Fetal Neonatal Ed. (2016) 101(5):F401–3. 10.1136/archdischild-2015-309328 26678879

[14] Hodgson KA Davis PG Owen LS. Nasal high flow therapy for neonates: current evidence and future directions. J Paediatr Child Health. (2019) 55(3):285–90. 10.1111/jpc.14374 30614098

[15] Spence KL Murphy D Kilian C McGonigle R Kilani RA. High-flow nasal cannula as a device to provide continuous positive airway pressure in infants. J Perinatol. (2007) 27(12):772–5. 10.1038/sj.jp.7211828 17762844

[16] Yengkhom R Suryawanshi P Gupta B Deshpande S. Heated humidified high-flow nasal cannula vs. Nasal continuous positive airway pressure for post-extubation respiratory support in preterm infants: a randomized controlled trial. J Trop Pediatr. (2021 Jan 29) 67(1):fmaa082. 10.1093/tropej/fmaa082 33174590

[17] Hasan RA Habib RH. Effects of flow rate and airleak at the nares and mouth opening on positive distending pressure delivery using commercially available high-flow nasal cannula systems: a lung model study. Pediatr Crit Care Med. (2011) 12(1):e29–33. 10.1097/PCC.0b013e3181d9076d 20228687

[18] Kubicka ZJ Limauro J Darnall RA. Heated, humidified high-flow nasal cannula therapy: yet another way to deliver continuous positive airway pressure? Pediatrics. (2008) 121(1):82–8. 10.1542/peds.2007-0957 18166560

[19] Dysart K Miller TL Wolfson MR Shaffer TH. Research in high flow therapy: mechanisms of action. Respir Med. (2009) 103(10):1400–5. 10.1016/j.rmed.2009.04.007 19467849

[20] Chao KY Chen YL Tsai LY Chien YH Mu SC. The role of heated humidified high-flow nasal cannula as noninvasive respiratory support in neonates. Pediatr Neonatol. (2017) 58(4):295–302. 10.1016/j.pedneo.2016.08.007 28223010

[21] Pediatrics ECoCJo. Neonatal mechanical ventilation routine. Chin J Pediatr. (2015) 53(5):4. 10.3760/cma.j.issn.0578-1310.2015.05.003

[22] Sweet DG Carnielli V Greisen G Hallman M Ozek E Plavka R European Consensus guidelines on the management of respiratory distress syndrome - 2016 update. Neonatology. (2017) 111(2):107–25. 10.1159/000448985 27649091

[23] Lavizzari A Colnaghi M Ciuffini F Veneroni C Musumeci S Cortinovis I Heated, humidified high-flow nasal cannula vs nasal continuous positive airway pressure for respiratory distress syndrome of prematurity: a randomized clinical noninferiority trial. JAMA Pediatr. (2016). 10.1001/jamapediatrics.2016.1243. [Online ahead of print] 27532363

[24] Chen J Gao WW Xu F Du LL Zhang T Ling X Comparison of clinical efficacy of heated humidified high flow nasal cannula versus nasal continuous positive airway pressure in treatment of respiratory distress syndrome in very low birth weight infants. Zhongguo Dang Dai Er Ke Za Zhi. (2015) 17(8):847–51. 10.7499/j.issn.1008-8830.2015.08.017 26287351

[25] Demirel G Vatansever B Tastekin A. High flow nasal cannula versus nasal continuous positive airway pressure for primary respiratory support in preterm infants: a prospective randomized study. Am J Perinatol. (2021) 38(3):237–41. 10.1055/s-0039-1696673 31563133

[26] Kotecha SJ Adappa R Gupta N Watkins WJ Kotecha S Chakraborty M. Safety and efficacy of high-flow nasal cannula therapy in preterm infants: a meta-analysis. Pediatrics. (2015) 136(3):542–53. 10.1542/peds.2015-0738 26283781

[27] Wilkinson D Andersen C O'Donnell CP De Paoli AG Manley BJ. High flow nasal cannula for respiratory support in preterm infants. Cochrane Database Syst Rev. (2016) 2:Cd006405. 10.1002/14651858.CD006405.pub3 26899543 PMC9371597

[28] Yoder BA Stoddard RA Li M King J Dirnberger DR Abbasi S. Heated, humidified high-flow nasal cannula versus nasal CPAP for respiratory support in neonates. Pediatrics. (2013) 131(5):e1482–90. 10.1542/peds.2012-2742 23610207

[29] Fleeman N Dundar Y Shah PS Shaw BN. Heated humidified high-flow nasal cannula for preterm infants: an updated systematic review and meta-analysis. Int J Technol Assess Health Care. (2019) 35(4):298–306. 10.1017/S0266462319000424 31292014

[30] Shin J Park K Lee EH Choi BM. Humidified high flow nasal cannula versus nasal continuous positive airway pressure as an initial respiratory support in preterm infants with respiratory distress: a randomized, controlled non-inferiority trial. J Korean Med Sci. (2017) 32(4):650–5. 10.3346/jkms.2017.32.4.650 28244292 PMC5334164

[31] Roberts CT Owen LS Manley BJ Frøisland DH Donath SM Dalziel KM Nasal high-flow therapy for primary respiratory support in preterm infants. N Engl J Med. (2016) 375(12):1142–51. 10.1056/NEJMoa1603694 27653564

[32] Hong H Li XX Li J Zhang ZQ. High-flow nasal cannula versus nasal continuous positive airway pressure for respiratory support in preterm infants: a meta-analysis of randomized controlled trials. J Matern Fetal Neonatal Med. (2021) 34(2):259–66. 10.1080/14767058.2019.1606193 30966839

[33] Conte F Orfeo L Gizzi C Massenzi L Fasola S. Rapid systematic review shows that using a high-flow nasal cannula is inferior to nasal continuous positive airway pressure as first-line support in preterm neonates. Acta Paediatrica. (2018) 107(10):1684–96. 10.1111/apa.14396 29751368

[34] Bruet S Butin M Dutheil F. Systematic review of high-flow nasal cannula versus continuous positive airway pressure for primary support in preterm infants. Arch Dis Child Fetal Neonatal Ed. (2022) 107(1):56–9. 10.1136/archdischild-2020-321094 34016651

[35] Hutton B Salanti G Caldwell DM Chaimani A Schmid CH Cameron C The PRISMA extension statement for reporting of systematic reviews incorporating network meta-analyses of health care interventions: checklist and explanations. Ann Intern Med. (2015) 162(11):777–84. 10.7326/M14-2385 26030634

[36] Keren L Jun T. High-flow nasal cannula versus continuous positive airway pressure for primary respiratory support in preterm infants: a systematic review and meta-analysis.

[37] Armanian AM Iranpour R Parvaneh M Salehimehr N Feizi A Hajirezaei M. Heated humidified high flow nasal cannula (HHHFNC) is not an effective method for initial treatment of respiratory distress syndrome (RDS) versus nasal intermittent mandatory ventilation (NIMV) and nasal continuous positive airway pressure (NCPAP). J Res Med Sci. (2019) 24:73. 10.4103/jrms.JRMS_2_19 31523259 PMC6734667

[38] Ciuffini F Pietrasanta C Lavizzari A Musumeci S Gualdi C Sortino S Comparison between two different modes of non-invasive ventilatory support in preterm newborn infants with respiratory distress syndrome mild to moderate: preliminary data. Pediatr Med Chir. (2014) 36(4):88. 10.4081/pmc.2014.88 25573704

[39] Farhat AS Mohammadzadeh A Mamuri GA Saeidi R Orizadeh SN. Comparison of nasal non-invasive ventilation methods in preterm neonates with respiratory distress syndrome. Iran J Neonatol. (2018) 9(4):53–60. 10.22038/ijn.2018.24544.1313

[40] Lin F Yan L Danhua M Hongjuan B Lipin Y Jin X Clinical trial on the effectiveness and safety of high flow nasal cannula oxygen therapy in preterm infants. Chinese J Clin Nutr. (2016) 3(26):2.

[41] Kadivar M Mosayebi Z Razi N Nariman S Sangsari R. High flow nasal cannulae versus nasal continuous positive airway pressure in neonates with respiratory distress syndrome managed with INSURE method: a randomized clinical trial. Iran J Med Sci. (2016) 41(6):494. PMID: 27853329 27853329 PMC5106564

[42] Li Wenying QA Xiaojiao W Yuanyuan W. Clinical observation on RDS treated with three kinds of auxiliary ventilation combined with pulmonary surfactant in low weight premature infants. J Pediatr Pharmacol. (2014) 07(20):4. 10.13407/j.cnki.jpp.1672-108X.2014.07.007

[43] Manley BJ Arnolda GRB Wright IMR Owen LS Foster JP Huang L Nasal high-flow therapy for newborn infants in special care nurseries. N Engl J Med. (2019) 380(21):2031–40. 10.1056/NEJMoa1812077 31116919

[44] Mostafa-Gharehbaghi M Mojabi H. Comparing the effectiveness of nasal continuous positive airway pressure (NCPAP) and high flow nasal cannula (HFNC) in prevention of post extubation assisted ventilation. Zahedan J Res Med Sci. (2015) 17(6):e984. 10.17795/zjrms984

[45] Murki S Singh J Khant C Kumar Dash S Oleti TP Joy P High-Flow nasal cannula versus nasal continuous positive airway pressure for primary respiratory support in preterm infants with respiratory distress: a randomized controlled trial. Neonatology. (2018) 113(3):235–41. 10.1159/000484400 29393237

[46] Öktem A Yiğit Ş Çelik HT Yurdakök M. Comparison of four different non-invasive respiratory support techniques as primary respiratory support in preterm infants. Turk J Pediatr. (2021) 63(1):23–30. 10.24953/turkjped.2021.01.003 33686823

[47] Sharma PK Poonia AK Bansal RK. Comparison of efficacy of nasal continuous positive airway pressure and heated humidified high-flow nasal cannula as a primary mode of respiratory support in preterm infants. J Clin Neonatol. (2019) 8(2):102. 10.4103/jcn.JCN_116_18

[48] Shirvani TE Nayeri FS Shariat M Nafs NN Mirjalili MR Hosseini SN Continuous positive airway pressure or humidified high flow nasal cannula for respiratory distress syndrome: a randomized control trial among premature neonates. Iran J Neonatol. (2020) 11(4):50–6. 10.22038/ijn.2020.46421.1783

[49] Shokouhi M Basiri B Sabzehei MK Mandiankhoo M Pirdehghan A. Efficacy and complications of humidified high-flow nasal cannula versus nasal continuous positive airway pressure in neonates with respiratory distress syndrome after surfactant therapy. Iran Red Crescent Med J. (2019) 21(2):e83615. 10.5812/ircmj.83615

[50] Yue W Weiwei W Yunbin C Xiuzhen Y Yong Z Fang L. Early HHFNC versus nCPAP in very low birth weight preterm infants. Chin J Women Child Health. (2013) 4(4):13–14. 10.19757/j.cnki.issn1674-7763.2013.z1.005

[51] Jing W Xiaoli W Junli L Yan W Guo Y Baohai S. Effect of heated humidified high flow nasal cannula in treatment for neonatal respiratory distress syndrome. Chin Med Eng. (2021) 3(29):6. 10.19338/j.issn.1672-2019.2021.03.019

[52] YAN Hui-yu Y-h. Value analysis of warming and humidifying high-flow nasal catheter ventilation in the treatment of respiratory distress syndrome in premature infants. World J Complex Med. (2020) 6(6):3. 10.11966/j.issn.2095-994X.2020.06.06.32

[53] YAO Fan XB Shisi LIN Qin LI. Comparison of clinical curative effects of nasal continuous positive airway pressure ventilation and humidified high flow nasal cannula in treatment of neonatal respiratory distress syndrome. Chin Med Pharm. (2019) 22(9):4. 10.3969/j.issn.2095-0616.2019.22.023

[54] Xiao-ping Y. Clinical analysis of different auxiliary ventilation methods to prevent extubation failure in very low birth weight premature infants. Smart Healthcare. (2018) 4(14):3. 10.19335/j.cnki.2096-1219.2018.14.017

[55] Zhai Jingfang WJ Bao J Xiao L Yanbo W Guangling Z Xiaoyu S Comparison of heated humidified high flow nasal cannula and nasal continuous positive airway pressure in initial respiratory support of mild neonatal respiratory distress syndrome. Chin J Obstet Gynecol Pediatr. (2019) 6(15):7. 10.3877/cma.j.issn.1673-5250.2019.06.005

[56] Ji-hua YJ-m ZHANG Yu-hong DING. Study on the efficacy of application of two kinds of auxiliary ventilation in treatment of respiratory distress syndrome in neonates with very low birth weight. J Clin Exp Med. (2017) 16(23):4. 10.3969/j.issn.1671-4695.2017.23.028

[57] Zhihua Z. Application of two kinds of non-invasive positive pressure ventilation in the treatment of neonatal respiratory distress syndrome. Med Inno of Chin. (2019) 16(20):4. 10.3969/j.issn.1674-4985.2019.20.031

[58] Jeon GW. Respiratory support with heated humidified high flow nasal cannula in preterm infants. Korean J Pediatr. (2016) 59(10):389–94. 10.3345/kjp.2016.59.10.389 27826324 PMC5099285

[59] Lampland AL Plumm B Meyers PA Worwa CT Mammel MC. Observational study of humidified high-flow nasal cannula compared with nasal continuous positive airway pressure. J Pediatr. (2009) 154(2):177–82. 10.1016/j.jpeds.2008.07.021 18760803

[60] Frizzola M Miller TL Rodriguez ME Zhu Y Rojas J Hesek A High-flow nasal cannula: impact on oxygenation and ventilation in an acute lung injury model. Pediatr Pulmonol. (2011) 46(1):67–74. 10.1002/ppul.21326 21171186 PMC3332105

