# Supplementary material for: High-flow nasal cannula versus continuous positive airway pressure in primary respiratory support for preterm infants: A systematic review and meta-analysis
Source: Front Pediatr. 2022 Nov 21;10:980024. doi: 10.3389/fped.2022.980024 (PMC9720183; doi:10.3389/fped.2022.980024)
Supplement: Supplementary file 1 [file Datasheet1.zip › Supplementary Materials/Appendix 1 Search strategy.pdf]

Pubmed		
#	Query	Results
1	"extremely premature infant"[Title/Abstract] OR "infant, extremely premature"[Title/Abstract] OR "Infant, Premature"[Title/Abstract] OR "infant, premature, diseases"[Title/Abstract] OR "Infant, Preterm"[Title/Abstract] OR "Infants, Premature"[Title/Abstract] OR "Infants, Preterm"[Title/Abstract] OR "Neonatal Prematurity"[Title/Abstract] OR "neonate, premature"[Title/Abstract] OR "premature"[Title/Abstract] OR "premature baby"[Title/Abstract] OR "premature birth"[Title/Abstract] OR "premature child"[Title/Abstract] OR "premature childbirth"[Title/Abstract] OR "Premature Infant"[Title/Abstract] OR "pre-mature infant"[Title/Abstract] OR "premature infant disease"[Title/Abstract] OR "premature infant diseases"[Title/Abstract] OR "Premature Infants"[Title/Abstract] OR "premature neonate"[Title/Abstract] OR "premature newborn"[Title/Abstract] OR "premature syndrome"[Title/Abstract] OR "prematuritas"[Title/Abstract] OR "prematurity"[Title/Abstract] OR "Prematurity, Neonatal"[Title/Abstract] OR "preterm baby"[Title/Abstract] OR "pre-term baby"[Title/Abstract] OR "preterm child"[Title/Abstract] OR "pre-term child"[Title/Abstract] OR "Preterm Infant"[Title/Abstract] OR "pre-term infant"[Title/Abstract] OR "Preterm Infants"[Title/Abstract] OR "preterm neonate"[Title/Abstract] OR "pre-term neonate"[Title/Abstract] OR "preterm newborn"[Title/Abstract] OR "pre-term newborn"[Title/Abstract]	173082
	"extremely premature infant"[Title/Abstract] OR "infant extremely	

	premature"[Title/Abstract] OR "infant premature"[Title/Abstract] OR "infant premature diseases"[Title/Abstract] OR "infant preterm"[Title/Abstract] OR "infants premature"[Title/Abstract] OR "infants preterm"[Title/Abstract] OR "Neonatal Prematurity"[Title/Abstract] OR "premature"[Title/Abstract] OR "premature baby"[Title/Abstract] OR "premature birth"[Title/Abstract] OR "premature child"[Title/Abstract] OR "premature childbirth"[Title/Abstract] OR "Premature Infant"[Title/Abstract] OR "pre-mature infant"[Title/Abstract] OR "premature infant disease"[Title/Abstract] OR "premature infant diseases"[Title/Abstract] OR "Premature Infants"[Title/Abstract] OR "premature neonate"[Title/Abstract] OR "premature newborn"[Title/Abstract] OR "premature syndrome"[Title/Abstract] OR "prematuritas"[Title/Abstract] OR "prematurity"[Title/Abstract] OR "prematurity neonatal"[Title/Abstract] OR "preterm baby"[Title/Abstract] OR "pre-term baby"[Title/Abstract] OR "preterm child"[Title/Abstract] OR "pre-term child"[Title/Abstract] OR "Preterm Infant"[Title/Abstract] OR "pre-term infant"[Title/Abstract] OR "Preterm Infants"[Title/Abstract] OR "preterm neonate"[Title/Abstract] OR "pre-term neonate"[Title/Abstract] OR "preterm newborn"[Title/Abstract] OR "pre-term newborn"[Title/Abstract]	
2	Infant, Premature[MeSH Terms]	61096
3	#1 OR #2	193765
4	"Cannula"[Title/Abstract] OR "Cannula, Nasal"[Title/Abstract] OR "Cannulae"[Title/Abstract] OR "Cannulae, Nasal"[Title/Abstract] OR "HF oxygen therapy"[Title/Abstract] OR "HFNC (high flow nasal cannula)"[Title/Abstract] OR "HFNC assisted ventilation"[Title/Abstract] OR "HFNC therapy"[Title/Abstract] OR "HFNC ventilation"[Title/Abstract]	21441

	OR "HFNCT (high flow nasal cannula therapy)"[Title/Abstract] OR "high flow nasal cannula"[Title/Abstract] OR "high flow nasal cannula respiratory support"[Title/Abstract] OR "high flow nasal cannula therapy"[Title/Abstract] OR "high flow nasal canula"[Title/Abstract] OR "high flow nasal prong therapy"[Title/Abstract] OR "high flow nasal therapy"[Title/Abstract] OR "high flow oxygenation therapy"[Title/Abstract] OR "high-flow (HF) oxygen therapy"[Title/Abstract] OR "highflow nasal cannula"[Title/Abstract] OR "highflow nasal cannula therapy"[Title/Abstract] OR "high-flow oxygen therapy"[Title/Abstract] OR "high-flow oxygen treatment"[Title/Abstract] OR "Nasal Cannula"[Title/Abstract] OR "Nasal Cannulae"[Title/Abstract] OR "nasal high flow"[Title/Abstract]	
5	Cannula[MeSH Terms]	1396
6	#4 OR #5	21610
7	"Airway Pressure Release Ventilation"[Title/Abstract] OR "APRV Ventilation Mode"[Title/Abstract] OR "APRV Ventilation Modes"[Title/Abstract] OR "Bilevel Continuous Positive Airway Pressure"[Title/Abstract] OR "Bilevel Positive Airway Pressure"[Title/Abstract] OR "BiPAP Bilevel Positive Airway Pressure"[Title/Abstract] OR "BiPAP Biphasic Positive Airway Pressure"[Title/Abstract] OR "Biphasic Continuous Positive Airway Pressure"[Title/Abstract] OR "Biphasic Positive Airway Pressure"[Title/Abstract] OR "constant positive airway pressure"[Title/Abstract] OR "constant positive pressure breathing"[Title/Abstract] OR "constant positive pressure ventilation"[Title/Abstract] OR "continous positive airway pressure"[Title/Abstract] OR "continuous positive airway pressure	11998

	ventilation"[Title/Abstract] OR "continuous positive pressure breathing"[Title/Abstract] OR "continuous positive pressure ventilation"[Title/Abstract] OR "CPAP"[Title/Abstract] OR "CPAP Ventilation"[Title/Abstract] OR "CPPB"[Title/Abstract] OR "CPPV"[Title/Abstract] OR "Nasal Continuous Positive Airway Pressure"[Title/Abstract] OR "nCPAP Ventilation"[Title/Abstract] OR "Ventilation Mode, APRV"[Title/Abstract] OR "Ventilation Modes, APRV"[Title/Abstract] OR "Ventilation, CPAP"[Title/Abstract] OR "Ventilation, nCPAP"[Title/Abstract]	
8	Continuous Positive Airway Pressure[MeSH Terms]	8385
9	#7 OR #8	15073
10	#3 AND #6 AND #9	245

Embase		
#	Query	Results
1	'extremely premature infant':ab,ti,kw OR 'infant, extremely premature':ab,ti,kw OR 'infant, premature':ab,ti,kw OR 'infant, premature, diseases':ab,ti,kw OR 'infant, preterm':ab,ti,kw OR 'infants, premature':ab,ti,kw OR 'infants, preterm':ab,ti,kw OR 'neonatal prematurity':ab,ti,kw OR 'neonate, premature':ab,ti,kw OR 'premature':ab,ti,kw OR 'premature baby':ab,ti,kw OR 'premature birth':ab,ti,kw OR 'premature child':ab,ti,kw OR 'premature childbirth':ab,ti,kw OR 'premature infant':ab,ti,kw OR 'pre-mature infant':ab,ti,kw OR 'premature infant disease':ab,ti,kw OR 'premature	234046

	infant diseases':ab,ti,kw OR 'premature infants':ab,ti,kw OR 'premature neonate':ab,ti,kw OR 'premature newborn':ab,ti,kw OR 'premature syndrome':ab,ti,kw OR 'prematuritas':ab,ti,kw OR 'prematurity':ab,ti,kw OR 'prematurity, neonatal':ab,ti,kw OR 'preterm baby':ab,ti,kw OR 'pre-term baby':ab,ti,kw OR 'preterm child':ab,ti,kw OR 'pre-term child':ab,ti,kw OR 'preterm infant':ab,ti,kw OR 'pre-term infant':ab,ti,kw OR 'preterm infants':ab,ti,kw OR 'preterm neonate':ab,ti,kw OR 'pre-term neonate':ab,ti,kw OR 'preterm newborn':ab,ti,kw OR 'pre-term newborn':ab,ti,kw	
2	'prematurity'/exp	123104
3	#1 OR #2	277970
4	'cannula':ti,ab,kw OR 'cannula, nasal':ti,ab,kw OR 'cannulae':ti,ab,kw OR 'cannulae, nasal':ti,ab,kw OR 'hf oxygen therapy':ti,ab,kw OR 'hfnc (high flow nasal cannula)':ti,ab,kw OR 'hfnc assisted ventilation':ti,ab,kw OR 'hfnc therapy':ti,ab,kw OR 'hfnc ventilation':ti,ab,kw OR 'hfnct (high flow nasal cannula therapy)':ti,ab,kw OR 'high flow nasal cannula':ti,ab,kw OR 'high flow nasal cannula respiratory support':ti,ab,kw OR 'high flow nasal cannula therapy':ti,ab,kw OR 'high flow nasal canula':ti,ab,kw OR 'high flow nasal prong therapy':ti,ab,kw OR 'high flow nasal therapy':ti,ab,kw OR 'high flow oxygenation therapy':ti,ab,kw OR 'high-flow (hf) oxygen therapy':ti,ab,kw OR 'highflow nasal cannula':ti,ab,kw OR 'highflow nasal cannula therapy':ti,ab,kw OR 'high-flow oxygen therapy':ti,ab,kw OR 'high-flow oxygen treatment':ti,ab,kw OR 'nasal cannula':ti,ab,kw OR 'nasal cannulae':ti,ab,kw OR 'nasal high flow':ti,ab,kw	31565
5	'high flow nasal cannula therapy'/exp	3025

6	#4 OR #5	32895
7	'airway pressure release ventilation':ti,ab,kw OR 'aprv ventilation mode':ti,ab,kw OR 'aprv ventilation modes':ti,ab,kw OR 'bilevel continuous positive airway pressure':ti,ab,kw OR 'bilevel positive airway pressure':ti,ab,kw OR 'bipap bilevel positive airway pressure':ti,ab,kw OR 'bipap biphasic positive airway pressure':ti,ab,kw OR 'biphasic continuous positive airway pressure':ti,ab,kw OR 'biphasic positive airway pressure':ti,ab,kw OR 'constant positive airway pressure':ti,ab,kw OR 'constant positive pressure breathing':ti,ab,kw OR 'constant positive pressure ventilation':ti,ab,kw OR 'continous positive airway pressure':ti,ab,kw OR 'continuous positive airway pressure ventilation':ti,ab,kw OR 'continuous positive pressure breathing':ti,ab,kw OR 'continuous positive pressure ventilation':ti,ab,kw OR 'cpap':ti,ab,kw OR 'cpap ventilation':ti,ab,kw OR 'cppb':ti,ab,kw OR 'cppv':ti,ab,kw OR 'nasal continuous positive airway pressure':ti,ab,kw OR 'ncpap ventilation':ti,ab,kw OR 'ventilation mode, aprv':ti,ab,kw OR 'ventilation modes, aprv':ti,ab,kw OR 'ventilation, cpap':ti,ab,kw OR 'ventilation, ncpap':ti,ab,kw	21939
8	'continuous positive airway pressure'/exp	5171
9	#7 OR #8	24712
10	#3 AND #6 AND #9	387

Cochrane Library

#	Query	Results
1	MeSH descriptor: [Infant, Premature] this term only	3901
2	(“extremely premature infant” or “infant, extremely premature” or “Infant, Premature” or “infant, premature, diseases” or “Infant, Preterm” or “Infants, Premature” or “Infants, Preterm” or “Neonatal Prematurity” or “neonate, premature” or “premature” or “premature baby” or “premature birth” or “premature child” or “premature childbirth” or “Premature Infant” or “pre-mature infant” or “premature infant disease” or “premature infant diseases” or “Premature Infants” or “premature neonate” or “premature newborn” or “premature syndrome” or “prematuritas” or “prematurity” or “Prematurity, Neonatal” or “preterm baby” or “pre-term baby” or “preterm child” or “pre-term child” or “Preterm Infant” or “pre-term infant” or “Preterm Infants” or “preterm neonate” or “pre-term neonate” or “preterm newborn” or “pre-term newborn”):ti,ab,kw	24558
3	#1 OR #2	24558
4	MeSH descriptor: [Cannula] this term only	153
5	(“Cannula” OR “Cannula, Nasal” OR “Cannulae” OR “Cannulae, Nasal” OR “HF oxygen therapy” OR “HFNC (high flow nasal cannula)” OR “HFNC assisted ventilation” OR “HFNC therapy” OR “HFNC ventilation” OR “HFNCT (high flow nasal cannula therapy)” OR “high flow nasal cannula” OR “high flow nasal cannula respiratory support” OR “high flow nasal cannula therapy” OR “high flow nasal canula” OR “high flow nasal prong therapy” OR	3905

	"high flow nasal therapy" OR "high flow oxygenation therapy" OR "high-flow (HF) oxygen therapy" OR "highflow nasal cannula" OR "highflow nasal cannula therapy" OR "high-flow oxygen therapy" OR "high-flow oxygen treatment" OR "Nasal Cannula" OR "Nasal Cannulae" OR "nasal high flow"):ti,ab,kw	
6	#4 OR #5	3905
7	MeSH descriptor: [Continuous Positive Airway Pressure] this term only	1238
8	("Airway Pressure Release Ventilation" OR "APRV Ventilation Mode" OR "APRV Ventilation Modes" OR "Bilevel Continuous Positive Airway Pressure" OR "Bilevel Positive Airway Pressure" OR "BiPAP Bilevel Positive Airway Pressure" OR "BiPAP Biphasic Positive Airway Pressure" OR "Biphasic Continuous Positive Airway Pressure" OR "Biphasic Positive Airway Pressure" OR "constant positive airway pressure" OR "constant positive pressure breathing" OR "constant positive pressure ventilation" OR "continous positive airway pressure" OR "continuous positive airway pressure ventilation" OR "continuous positive pressure breathing" OR "continuous positive pressure ventilation" OR "CPAP" OR "CPAP Ventilation" OR "CPPB" OR "CPPV" OR "Nasal Continuous Positive Airway Pressure" OR "nCPAP Ventilation" OR "Ventilation Mode, APRV" OR "Ventilation Modes, APRV" OR "Ventilation, CPAP" OR "Ventilation, nCPAP"):ti,ab,kw	5631
9	#7 OR #8	5832
10	#3 AND #6 AND #9 in Trials	212

CNKI		
#	Query	Results
1	(全文=(('HFNC' + '高流量' + '经鼻高流量') * ('CPAP' + '持续正压'))) AND (篇关摘=('早产' + '低出生体重'))	124

VIP		
#	Query	Results
1	M=(((("HFNC" OR "高流量" OR "经鼻高流量") AND ("CPAP" OR "持续正压"))) AND ("早产" OR "低出生体重")) NOT ("meta" OR "系统评价")	43

WANFANG		
#	Query	Results
1	主题=(((("HFNC" OR "高流量" OR "经鼻高流量") AND ("CPAP" OR "持续正压"))) AND ("早产" OR "低出生体重")) NOT ("meta" OR "系统评价")	33

SinoMed		
#	Query	Results
1	("HFNC" OR "高流量") AND ("CPAP" OR "持续正压") AND ("早产" OR "	27

	低出生体重")	
