# Supplementary material for: High-flow nasal cannula versus continuous positive airway pressure in primary respiratory support for preterm infants: A systematic review and meta-analysis
Source: Front Pediatr. 2022 Nov 21;10:980024. doi: 10.3389/fped.2022.980024 (PMC9720183; doi:10.3389/fped.2022.980024)
Supplement: Supplementary file 1 [file Datasheet1.zip › Supplementary Materials/Appendix 2 Risk of bias table of included studies..pdf]

Characteristics of studies

Characteristics of included studies

Armanian 2019

**Methods**	
**Participants**	
**Interventions**	
**Outcomes**	
**Notes**	

Risk of bias table

**Bias**	**Authors' judgement**	**Support for judgement**
Random sequence generation (selection bias)	Low risk	The neonates were randomly assigned into the study groups. Due to the gradual enrollment of patients into the study, the randomized assignment was performed using envelopes containing names of the therapy group using permuted block randomization of size 6.
Allocation concealment (selection bias)	Low risk	Patients are all infants.
Blinding of participants and personnel (performance bias)	High risk	Unable to be blinded.
Blinding of outcome assessment (detection bias)	High risk	Unable to be blinded.
Incomplete outcome data (attrition bias)	Low risk	All included objetcs were included in the analysis of primary outcomes.
Selective reporting (reporting bias)	Low risk	"The current study was registered in the Iranian Registry of Clinical Trials (IRCT2016052510026N7)."<br>The protocol is achievable and the report follows the protocol perfectly.
Other bias	Low risk	Not found.

Chen 2015

**Methods**	
**Participants**	
**Interventions**	
**Outcomes**	

**Notes**	

Risk of bias table

Bias	Authors' judgement	Support for judgement
Random sequence generation (selection bias)	Unclear risk	Randomization grouping is not described in detail.
Allocation concealment (selection bias)	Low risk	Patients are all infants.
Blinding of participants and personnel (performance bias)	High risk	Unable to be blinded.
Blinding of outcome assessment (detection bias)	High risk	Unable to be blinded.
Incomplete outcome data (attrition bias)	Low risk	All included objetcs were included in the analysis of primary outcomes.
Selective reporting (reporting bias)	Unclear risk	The protocol is not found.
Other bias	Low risk	Not found.

Ciuffini 2014

**Methods**	
**Participants**	
**Interventions**	
**Outcomes**	
**Notes**	

Risk of bias table

Bias	Authors' judgement	Support for judgement
Random sequence generation (selection bias)	Low risk	The infants were randomized into two groups, with random randomization in blocks (29-32, 33-34 and 35-36 weeks of gestational age), using the method of closed envelopes.
Allocation concealment (selection bias)	Low risk	Patients are all infants.
Blinding of participants and personnel (performance bias)	High risk	Unable to be blinded.
Blinding of outcome assessment (detection bias)	High risk	Unable to be blinded.

Incomplete outcome data (attrition bias)	Low risk	All included objetcs were included in the analysis of primary outcomes.
Selective reporting (reporting bias)	Unclear risk	The protocol is not found.
Other bias	Low risk	Not found.

Demirel 2019

**Methods**	
**Participants**	
**Interventions**	
**Outcomes**	
**Notes**	

Risk of bias table

Bias	Authors' judgement	Support for judgement
Random sequence generation (selection bias)	Low risk	Randomization was computer generated, performed by using random number, and sequentially numbered sealed opaque envelopes containing group assignments were prepared.
Allocation concealment (selection bias)	Low risk	Patients are all infants.
Blinding of participants and personnel (performance bias)	High risk	Unable to be blinded.
Blinding of outcome assessment (detection bias)	High risk	Unable to be blinded.
Incomplete outcome data (attrition bias)	Low risk	All included objetcs were included in the analysis of primary outcomes.
Selective reporting (reporting bias)	Unclear risk	The protocol is not found.
Other bias	Low risk	Not found.

Farhat 2018

**Methods**	
**Participants**	
**Interventions**	
**Outcomes**	

**Notes**	

Risk of bias table

Bias	Authors' judgement	Support for judgement
Random sequence generation (selection bias)	Low risk	All the neonates entering the study were randomized groups matched for birth weight and gestational age.
Allocation concealment (selection bias)	Low risk	Patients are all infants.
Blinding of participants and personnel (performance bias)	High risk	Unable to be blinded.
Blinding of outcome assessment (detection bias)	High risk	Unable to be blinded.
Incomplete outcome data (attrition bias)	Low risk	All included objetcs were included in the analysis of primary outcomes.
Selective reporting (reporting bias)	Unclear risk	The protocol is not found.
Other bias	Low risk	Not found.

Feng 2016

**Methods**	
**Participants**	
**Interventions**	
**Outcomes**	
**Notes**	

Risk of bias table

Bias	Authors' judgement	Support for judgement
Random sequence generation (selection bias)	Unclear risk	Randomization grouping is not described in detail.
Allocation concealment (selection bias)	Low risk	Patients are all infants.
Blinding of participants and personnel (performance bias)	High risk	Unable to be blinded.
Blinding of outcome assessment (detection bias)	High risk	Unable to be blinded.

Incomplete outcome data (attrition bias)	Low risk	All included objetcs were included in the analysis of primary outcomes.
Selective reporting (reporting bias)	Unclear risk	The protocol is not found.
Other bias	Low risk	Not found.

Kadivar 2016

**Methods**	
**Participants**	
**Interventions**	
**Outcomes**	
**Notes**	

Risk of bias table

**Bias**	**Authors' judgement**	**Support for judgement**
Random sequence generation (selection bias)	Low risk	Maternal characteristics (e.g. age), medical problems (e.g. diabetes or hypertension during pregnancy, gestational age, mode of delivery, prenatal corticosteroid administrations), and neonatal characteristics (e.g. birth weight, and sex of infants) were included by block randomized pilot study.
Allocation concealment (selection bias)	Low risk	Patients are all infants.
Blinding of participants and personnel (performance bias)	High risk	Unable to be blinded.
Blinding of outcome assessment (detection bias)	High risk	Unable to be blinded.
Incomplete outcome data (attrition bias)	Low risk	All included objetcs were included in the analysis of primary outcomes.
Selective reporting (reporting bias)	Unclear risk	The protocol is not found.
Other bias	Low risk	Not found.

Lavizzari 2016

**Methods**	
**Participants**	
**Interventions**	

**Outcomes**	
**Notes**	

Risk of bias table

Bias	Authors' judgement	Support for judgement
Random sequence generation (selection bias)	Low risk	Block randomization was applied, with a block size of 4. Infants were stratified according to GA: 29+0 to 32+6 weeks, 33+0 to 34+6 weeks, and 35+0 to 36+6 weeks. Infants born from multiple gestations were assigned by individual randomization.
Allocation concealment (selection bias)	Low risk	Patients are all infants.
Blinding of participants and personnel (performance bias)	High risk	Unable to be blinded.
Blinding of outcome assessment (detection bias)	High risk	Unable to be blinded.
Incomplete outcome data (attrition bias)	Low risk	All included objetcs were included in the analysis of primary outcomes.
Selective reporting (reporting bias)	Unclear risk	The protocol is not found.
Other bias	Low risk	Not found.

Li 2014

**Methods**	
**Participants**	
**Interventions**	
**Outcomes**	
**Notes**	

Risk of bias table

Bias	Authors' judgement	Support for judgement
Random sequence generation (selection bias)	Unclear risk	Randomization grouping is not described in detail.
Allocation concealment (selection bias)	Low risk	Patients are all infants.

Blinding of participants and personnel (performance bias)	High risk	Unable to be blinded.
Blinding of outcome assessment (detection bias)	High risk	Unable to be blinded.
Incomplete outcome data (attrition bias)	Low risk	All included objetcs were included in the analysis of primary outcomes.
Selective reporting (reporting bias)	Unclear risk	The protocol is not found.
Other bias	Low risk	Not found.

Manley 2019

**Methods**	
**Participants**	
**Interventions**	
**Outcomes**	
**Notes**	

Risk of bias table

**Bias**	**Authors' judgement**	**Support for judgement**
Random sequence generation (selection bias)	Low risk	A computer-generated randomization sequence with variable block sizes was used. Sequentially numbered, sealed, opaque envelopes containing the treatment assignment were opened.
Allocation concealment (selection bias)	Low risk	Patients are all infants.
Blinding of participants and personnel (performance bias)	High risk	Unable to be blinded.
Blinding of outcome assessment (detection bias)	High risk	Unable to be blinded.
Incomplete outcome data (attrition bias)	Low risk	All included objetcs were included in the analysis of primary outcomes.
Selective reporting (reporting bias)	Low risk	"The authors vouch for the accuracy and completeness of the data and for the fidelity of the trial to the protocol (published previously and available with the full text of this article at NEJM.org)."<br>The protocol is achievable and the report follows the protocol perfectly.
Other bias	Low risk	Not found.

Mostafa-Gharehbaghi 2015

**Methods**	
**Participants**	
**Interventions**	
**Outcomes**	
**Notes**	

Risk of bias table

Bias	Authors' judgement	Support for judgement
Random sequence generation (selection bias)	Low risk	Infants were randomly allocated in nasal CPAP or HFNC group according to random number list.
Allocation concealment (selection bias)	Low risk	Patients are all infants.
Blinding of participants and personnel (performance bias)	High risk	Unable to be blinded.
Blinding of outcome assessment (detection bias)	High risk	Unable to be blinded.
Incomplete outcome data (attrition bias)	Low risk	All included objetcs were included in the analysis of primary outcomes.
Selective reporting (reporting bias)	Low risk	"The study was registered in Iranian registry clinical trials (IRCT 201308283915 N9)."<br>The protocol is achievable and the report follows the protocol perfectly.
Other bias	Low risk	Not found.

Murki 2018

**Methods**	
**Participants**	
**Interventions**	
**Outcomes**	
**Notes**	

Risk of bias table

Bias	Authors' judgement	Support for judgement
Random sequence generation (selection bias)	Low risk	Randomization was stratified for SAS (>5 and ⩽5), gestation in weeks (34 weeks) and study center. Randomization was computer generated and group assignment was placed in serially numbered, opaque, sealed envelopes that were opened by research personnel after entering the baby details on the outside cover. Due to the nature of intervention, blinding was not attempted.
Allocation concealment (selection bias)	Low risk	Patients are all infants.
Blinding of participants and personnel (performance bias)	High risk	Unable to be blinded.
Blinding of outcome assessment (detection bias)	High risk	Unable to be blinded.
Incomplete outcome data (attrition bias)	Low risk	All included objetcs were included in the analysis of primary outcomes.
Selective reporting (reporting bias)	Low risk	"The study was registered prospectively in the clinical trial registry of India (CTRI/2015/08/006108)."<br>The protocol is achievable and the report follows the protocol perfectly.
Other bias	Low risk	Not found.

Öktem 2021

**Methods**	
**Participants**	
**Interventions**	
**Outcomes**	
**Notes**	

Risk of bias table

Bias	Authors' judgement	Support for judgement
Random sequence generation (selection bias)	Unclear risk	Randomization grouping is not described in detail.
Allocation concealment (selection bias)	Low risk	Patients are all infants.
Blinding of participants and personnel (performance bias)	High risk	Unable to be blinded.

Blinding of outcome assessment (detection bias)	High risk	Unable to be blinded.
Incomplete outcome data (attrition bias)	Low risk	All included objetcs were included in the analysis of primary outcomes.
Selective reporting (reporting bias)	Unclear risk	The protocol is not found.
Other bias	Low risk	Not found.

Roberts 2016

**Methods**	
**Participants**	
**Interventions**	
**Outcomes**	
**Notes**	

Risk of bias table

Bias	Authors' judgement	Support for judgement
Random sequence generation (selection bias)	Low risk	A computer-generated randomization sequence with variable block sizes was used. Infants were stratified according to gestational age and study center. Sequentially numbered, sealed, opaque envelopes containing the treatment assignment were opened.
Allocation concealment (selection bias)	Low risk	Patients are all infants.
Blinding of participants and personnel (performance bias)	High risk	Unable to be blinded.
Blinding of outcome assessment (detection bias)	High risk	Unable to be blinded.
Incomplete outcome data (attrition bias)	Low risk	All included objetcs were included in the analysis of primary outcomes.
Selective reporting (reporting bias)	Low risk	"All authors vouch for the accuracy and completeness of the data and for the fidelity of the study to the protocol, which was published previously and is available with the full text of this article at NEJM.org."<br>The protocol is achievable and the report follows the protocol perfectly.
Other bias	Low risk	Not found.

Sharma 2019

**Methods**	
**Participants**	
**Interventions**	
**Outcomes**	
**Notes**	

Risk of bias table

Bias	Authors' judgement	Support for judgement
Random sequence generation (selection bias)	Low risk	Randomization is conducted by using computer-generated random numbers.
Allocation concealment (selection bias)	Low risk	Patients are all infants.
Blinding of participants and personnel (performance bias)	High risk	Unable to be blinded.
Blinding of outcome assessment (detection bias)	High risk	Unable to be blinded.
Incomplete outcome data (attrition bias)	Low risk	All included objetcs were included in the analysis of primary outcomes.
Selective reporting (reporting bias)	Unclear risk	The protocol is not found.
Other bias	Low risk	Not found.

Shin 2017

**Methods**	
**Participants**	
**Interventions**	
**Outcomes**	
**Notes**	

Risk of bias table

Bias	Authors' judgement	Support for judgement
Random sequence generation (selection bias)	Low risk	Randomization was performed by using random-number, computer-generated randomization (Excel; Microsoft Corp., Redmond, WA, USA), and sequentially numbered sealed opaque envelopes that contained the group assignments

		were prepared.
Allocation concealment (selection bias)	Low risk	Patients are all infants.
Blinding of participants and personnel (performance bias)	High risk	Unable to be blinded.
Blinding of outcome assessment (detection bias)	High risk	Unable to be blinded.
Incomplete outcome data (attrition bias)	Low risk	All included objetcs were included in the analysis of primary outcomes.
Selective reporting (reporting bias)	Unclear risk	The protocol is not found.
Other bias	Low risk	Not found.

Shirvani 2020

**Methods**	
**Participants**	
**Interventions**	
**Outcomes**	
**Notes**	

Risk of bias table

**Bias**	**Authors' judgement**	**Support for judgement**
Random sequence generation (selection bias)	Unclear risk	Randomization grouping is not described in detail.
Allocation concealment (selection bias)	Low risk	Patients are all infants.
Blinding of participants and personnel (performance bias)	High risk	Unable to be blinded.
Blinding of outcome assessment (detection bias)	High risk	Unable to be blinded.
Incomplete outcome data (attrition bias)	Low risk	All included objetcs were included in the analysis of primary outcomes.
Selective reporting (reporting bias)	Unclear risk	The protocol is not found.
Other bias	Low risk	Not found.

Shokouhi 2019

**Methods**	
**Participants**	
**Interventions**	
**Outcomes**	
**Notes**	

Risk of bias table

Bias	Authors' judgement	Support for judgement
Random sequence generation (selection bias)	Low risk	Randomization is conducted by using the table of random numbers based on computer programs.
Allocation concealment (selection bias)	Low risk	Patients are all infants.
Blinding of participants and personnel (performance bias)	High risk	Unable to be blinded.
Blinding of outcome assessment (detection bias)	High risk	Unable to be blinded.
Incomplete outcome data (attrition bias)	Low risk	All included objetcs were included in the analysis of primary outcomes.
Selective reporting (reporting bias)	Low risk	"This study was registered at the Iranian Registry for Clinical Trials (Code: IRCT201709049014N181)."<br>The protocol is achievable and the report follows the protocol perfectly.
Other bias	Low risk	Not found.

Wang 2013

**Methods**	
**Participants**	
**Interventions**	
**Outcomes**	
**Notes**	

Risk of bias table

Bias	Authors' judgement	Support for judgement
Random sequence generation (selection bias)	Unclear risk	Randomization grouping is not described in detail.
Allocation concealment (selection bias)	Low risk	Patients are all infants.
Blinding of participants and personnel (performance bias)	High risk	Unable to be blinded.
Blinding of outcome assessment (detection bias)	High risk	Unable to be blinded.
Incomplete outcome data (attrition bias)	Low risk	All included objetcs were included in the analysis of primary outcomes.
Selective reporting (reporting bias)	Unclear risk	The protocol is not found.
Other bias	Low risk	Not found.

Wang 2021

**Methods**	
**Participants**	
**Interventions**	
**Outcomes**	
**Notes**	

Risk of bias table

Bias	Authors' judgement	Support for judgement
Random sequence generation (selection bias)	Low risk	Randomization is conducted by using the random number table.
Allocation concealment (selection bias)	Low risk	Patients are all infants.
Blinding of participants and personnel (performance bias)	High risk	Unable to be blinded.
Blinding of outcome assessment (detection bias)	High risk	Unable to be blinded.
Incomplete outcome data (attrition bias)	Low risk	All included objetcs were included in the analysis of primary outcomes.
Selective reporting (reporting bias)	Unclear risk	The protocol is not found.
Other bias	Low risk	Not found.

Yan 2020

**Methods**	
**Participants**	
**Interventions**	
**Outcomes**	
**Notes**	

Risk of bias table

Bias	Authors' judgement	Support for judgement
Random sequence generation (selection bias)	Low risk	Randomization is conducted by using the random number table.
Allocation concealment (selection bias)	Low risk	Patients are all infants.
Blinding of participants and personnel (performance bias)	High risk	Unable to be blinded.
Blinding of outcome assessment (detection bias)	High risk	Unable to be blinded.
Incomplete outcome data (attrition bias)	Low risk	All included objetcs were included in the analysis of primary outcomes.
Selective reporting (reporting bias)	Unclear risk	The protocol is not found.
Other bias	Low risk	Not found.

Yao 2019

**Methods**	
**Participants**	
**Interventions**	
**Outcomes**	
**Notes**	

Risk of bias table

Bias	Authors' judgement	Support for judgement
Random sequence generation (selection bias)	Low risk	Randomization is conducted by using the random number table.
Allocation concealment (selection bias)	Low risk	Patients are all infants.

Blinding of participants and personnel (performance bias)	High risk	Unable to be blinded.
Blinding of outcome assessment (detection bias)	High risk	Unable to be blinded.
Incomplete outcome data (attrition bias)	Low risk	All included objetcs were included in the analysis of primary outcomes.
Selective reporting (reporting bias)	Unclear risk	The protocol is not found.
Other bias	Low risk	Not found.

Yoder 2013

**Methods**	
**Participants**	
**Interventions**	
**Outcomes**	
**Notes**	

Risk of bias table

**Bias**	**Authors' judgement**	**Support for judgement**
Random sequence generation (selection bias)	Low risk	Infantswere randomly assigned at each study site via opaque sealed envelopes in blocks of 10 by study site by using random-number generation. Randomization was stratified by the following: birth weight and age.
Allocation concealment (selection bias)	Low risk	Patients are all infants.
Blinding of participants and personnel (performance bias)	High risk	Unable to be blinded.
Blinding of outcome assessment (detection bias)	High risk	Unable to be blinded.
Incomplete outcome data (attrition bias)	Low risk	All included objetcs were included in the analysis of primary outcomes.
Selective reporting (reporting bias)	Low risk	"This study was registered at clinicaltrials.gov (NCT00609882)."<br>The protocol is achievable and the report follows the protocol perfectly.
Other bias	Low risk	Not found.

Yu 2018

**Methods**	
**Participants**	
**Interventions**	
**Outcomes**	
**Notes**	

Risk of bias table

Bias	Authors' judgement	Support for judgement
Random sequence generation (selection bias)	Unclear risk	Randomization grouping is not described in detail.
Allocation concealment (selection bias)	Low risk	Patients are all infants.
Blinding of participants and personnel (performance bias)	High risk	Unable to be blinded.
Blinding of outcome assessment (detection bias)	High risk	Unable to be blinded.
Incomplete outcome data (attrition bias)	Low risk	All included objetcs were included in the analysis of primary outcomes.
Selective reporting (reporting bias)	Unclear risk	The protocol is not found.
Other bias	Low risk	Not found.

Zhai 2019

**Methods**	
**Participants**	
**Interventions**	
**Outcomes**	
**Notes**	

Risk of bias table

Bias	Authors' judgement	Support for judgement
Random sequence generation (selection bias)	Low risk	Randomization is conducted by using the random number table.
Allocation concealment (selection bias)	Low risk	Patients are all infants.

Blinding of participants and personnel (performance bias)	High risk	Unable to be blinded.
Blinding of outcome assessment (detection bias)	High risk	Unable to be blinded.
Incomplete outcome data (attrition bias)	Low risk	All included objetcs were included in the analysis of primary outcomes.
Selective reporting (reporting bias)	Unclear risk	The protocol is not found.
Other bias	Low risk	Not found.

Zhang 2017

**Methods**	
**Participants**	
**Interventions**	
**Outcomes**	
**Notes**	

Risk of bias table

**Bias**	**Authors' judgement**	**Support for judgement**
Random sequence generation (selection bias)	Low risk	Stratified randomization.
Allocation concealment (selection bias)	Low risk	Patients are all infants.
Blinding of participants and personnel (performance bias)	High risk	Unable to be blinded.
Blinding of outcome assessment (detection bias)	High risk	Unable to be blinded.
Incomplete outcome data (attrition bias)	Low risk	All included objetcs were included in the analysis of primary outcomes.
Selective reporting (reporting bias)	Unclear risk	The protocol is not found.
Other bias	Low risk	Not found.

Zhang 2019

**Methods**	
**Participants**	
**Interventions**	

**Outcomes**	
**Notes**	

Risk of bias table

**Bias**	**Authors' judgement**	**Support for judgement**
Random sequence generation (selection bias)	Low risk	Randomization is conducted by using the random number table.
Allocation concealment (selection bias)	Low risk	Patients are all infants.
Blinding of participants and personnel (performance bias)	High risk	Unable to be blinded.
Blinding of outcome assessment (detection bias)	High risk	Unable to be blinded.
Incomplete outcome data (attrition bias)	Low risk	All included objetcs were included in the analysis of primary outcomes.
Selective reporting (reporting bias)	Unclear risk	The protocol is not found.
Other bias	Low risk	Not found.

Footnotes
